# A *Drosophila *systems model of pentylenetetrazole induced locomotor plasticity responsive to antiepileptic drugs

**DOI:** 10.1186/1752-0509-3-11

**Published:** 2009-01-21

**Authors:** Farhan Mohammad, Priyanka Singh, Abhay Sharma

**Affiliations:** 1 Institute of Genomics and Integrative Biology, Mall Road, Delhi University Campus, Delhi 110007, India; 2 Tata Institute of Fundamental Research, Department of Biological Research, Dr. Homi Bhaba Road, Navy Nagar, Colaba, Mumbai-5, India

## Abstract

**Background:**

Rodent kindling induced by PTZ is a widely used model of epileptogenesis and AED testing. Overlapping pathophysiological mechanisms may underlie epileptogenesis and other neuropsychiatric conditions. Besides epilepsy, AEDs are widely used in treating various neuropsychiatric disorders. Mechanisms of AEDs' long term action in these disorders are poorly understood. We describe here a *Drosophila *systems model of PTZ induced locomotor plasticity that is responsive to AEDs.

**Results:**

We empirically determined a regime in which seven days of PTZ treatment and seven days of subsequent PTZ discontinuation respectively cause a decrease and an increase in climbing speed of *Drosophila *adults. Concomitant treatment with NaVP and LEV, not ETH, GBP and VGB, suppressed the development of locomotor deficit at the end of chronic PTZ phase. Concomitant LEV also ameliorated locomotor alteration that develops after PTZ withdrawal. Time series of microarray expression profiles of heads of flies treated with PTZ for 12 hrs (beginning phase), two days (latent phase) and seven days (behaviorally expressive phase) showed only down-, not up-, regulation of genes; expression of 23, 2439 and 265 genes were downregulated, in that order. GO biological process enrichment analysis showed downregulation of transcription, neuron morphogenesis during differentiation, synaptic transmission, regulation of neurotransmitter levels, neurogenesis, axonogenesis, protein modification, axon guidance, actin filament organization etc. in the latent phase and of glutamate metabolism, cell communication etc. in the expressive phase. Proteomic interactome based analysis provided further directionality to these events. Pathway overrepresentation analysis showed enrichment of Wnt signaling and other associated pathways in genes downregulated by PTZ. Mining of available transcriptomic and proteomic data pertaining to established rodent models of epilepsy and human epileptic patients showed overrepresentation of epilepsy associated genes in our PTZ regulated set.

**Conclusion:**

Systems biology ultimately aims at delineating and comprehending the functioning of complex biological systems in such details that predictive models of human diseases could be developed. Due to immense complexity of higher organisms, systems biology approaches are however currently focused on simpler organisms. Amenable to modeling, our model offers a unique opportunity to further dissect epileptogenesis-like plasticity and to unravel mechanisms of long-term action of AEDs relevant in neuropsychiatric disorders.

## Background

Epileptogenesis is poorly understood in cellular and molecular terms [1,2]. A systems level understanding of epileptogenesis – a network problem that may involve molecular, structural, and functional alterations in the brain – is expected to facilitate development of novel antiepileptogenic, disease-modifying, and neuroprotective agents [3]. Besides epilepsy, AEDs are also used in treating various other neurological and psychiatric conditions [4-6]. Overlapping pathophysiological mechanisms may underlie epilepsy and these other nonepileptic conditions [4,7]. Kindling is a model of brain plasticity in which recurrent activation of neural pathways results in an increased susceptibility to evoked seizures that ultimately progresses to spontaneous seizures [8]. Kindling in rodents is widely used to model epileptogenesis [1,2] – process whereby structural and functional changes occur following an insult that in some cases result in epilepsy and processes that contribute to the progression observed in some epilepsies including post-seizure cognitive and emotional deficits – and to test AEDs [9-13]. Not surprisingly, kindling-like phenomena is also considered relevant in various neuropsychiatric conditions [14-16]. Only a limited understanding exists at present as to how the initial electrographic seizure-induced changes in synaptic transmission and gene expression relate to permanent alteration in brain function induced by kindling [17].

Seizure in *Drosophila *and man have several similarities, and the utility of the fruit fly as a genetic model system for studying human seizure disorders and seizure-susceptibility has clearly been demonstrated [18-23]. *Drosophila *system has also been found useful in anticonvulsant drug screening and drug target identification [24-27]. The ultimate goal of systems biology is to delineate and to comprehend the functioning of complex biological systems in such details that predictive models of human diseases could be developed [28]. However, due to immense complexity of higher organisms, systems biology approaches are currently focused on developing simple organisms as integrative models [29,30]. For example, inherent complexity of mammalian brain however does not render the available rodent epilepsy models as amenable to systems modeling [3]. Given the above, we selected *Drosophila *for developing a first *ab initio *systems level model of brain plasticity that might be relevant in understanding mechanisms underlying epileptogenesis and AEDs' long-term therapeutic action in these neuropsychiatric conditions.

Rodent kindling induced by PTZ is the most popular pharmacologically induced kindling model that provides an acceptable approach for quantifying epileptogenesis and for testing AEDs [31]. In rodents, PTZ kindling is induced by repeated injection of a subconvulsant dose of the GABA antagonist over several weeks, resulting in partially and fully kindled animals with the latter group showing clonic-tonic seizures; once kindled, the state of behavioral hyperexcitability persists for up to several weeks after discontinuation of chemoconvulsant treatment [14,32]. Increased seizure susceptibility is known to occur via excitatory GABAergic signaling in *Drosophila *[19]. Moreover, chronic exposure to PTZ, a GABA antagonist, has been reported to cause seizure phenotypes in *Drosophila *larvae [21]. We thus selected PTZ for systems modeling. Altered locomotor activity is one of the kindling and postkindling behavior in rats and mice [33,34]. Also, seizure-like activity in *Drosophila *adults is known to be associated with loss of motor coordination, and altered locomotor activities [35]. Locomotor activity is a complex behavior and different neural systems may influence it in fly [36]. Locomotor behavior of *Drosophila *adults is used to model neuropsychiatric conditions [37]. Given the above, we selected *Drosophila *adults and focused on developing a locomotor behavior based model.

## Methods

### Fly culture and harvesting

*D. melanogaster *wild type Oregon-R strain was used. Routine cultures were maintained at 24 + 1°C, 60% RH, and 12 hrs light (9 AM to 9 PM) and 12 hrs dark cycle. Standard fly medium consisting of agar-agar, maize powder, brown sugar, dried yeast and nipagin was used. Standard methods of fly handling and manipulation were followed. Stringency required in behavioral studies was strictly adhered to at various levels, conditions of housing, exposure to anesthetic agent, light intensity, for example. Three to four days old unmated male flies were used to begin all experiments except dsRNA microinjection. For microinjection, 5–6 day old flies were used.

### Drug dosage and treatment

LC_50_, defined as concentration in normal fly media causing 50% lethality in seven days, was determined for PTZ and the AEDs (results not presented). Unless mentioned otherwise, half of LC_50_, 8, 3.48, 0.33 and 5 mg/ml for PTZ, ETH, NaVP (all from Sigma-Aldrich), and LEV (Levesam 500, Nicholas Piramal), respectively, was used. As GBP and VGB (both from Sigma-Aldrich) did not cause lethality up to 16 and 24 mg/ml respectively, the same concentrations were used in all the experiments. Thirty flies were housed in each treatment vial. Flies were maintained at 24 + 1°C, 60% RH, and 12 hrs light (9 AM to 9 PM) and 12 hrs dark cycle.

### Locomotor assays

Climbing speed was measured using an indigenously developed semi-manual method that was validated by DIAS (v. 3.4.2, Soll Technologies). In semi-manual assay, a glass column of 2 cm internal diameter and 30 cm internal length, i.e., length between the two cotton plugs, was used in the assays. The column was marked with lines at every cm along the length. Each fly was first familiarized in the vertically placed column for 90 seconds. The fly was thereafter brought to the bottom of the tube, by tapping the tube on a piece of packing foam. As soon as the fly had fallen on the cotton plug at the bottom, the tube was as such placed vertically and, in semi-manual method, a "dot/comma" recording, explained below, was applied the moment fly crossed a height of 5 cm. In "dot/comma" recording, the locomotor activity of a single fly was monitored by keeping pressed the dot key or the comma key of a personal computer, to record a moving or resting fly respectively. Dots and commas were subsequently transformed in activity and rest period respectively, using the cursor speed. Climbing speed was calculated using the following formula, *s *= *h*/*t*, where *s *= climbing speed, *h *= height climbed in cm, and *t *= activity period in sec. One fly each from different treatment groups was first scored as described below, discarded, and the exercise repeated. Measurements were taken at room temperature, between 9 AM to 9 PM. Climbing was considered complete when the fly reached the cotton plug at the top, fell to the bottom after climbing a certain height beyond 5 cm mark, or stopped after climbing to a certain height for more than 10 sec. Comma was applied only when the fly took rest. The vertical locomotor assay was adapted to measure horizontal (spontaneous) locomotor activities. A single fly was first brought to the middle of the column by gentle shaking and then the fly movement was constantly monitored for 90 sec by keeping pressed the dot key or the comma key of a personal computer, to record a moving or resting fly respectively. The total number of lines that a fly crossed in the 90 sec assay time was counted and noted down at the end of the dot/comma recording. The data was normalized for 90 sec and the dots and commas were subsequently transformed in rest and activity period respectively, using the cursor speed. Walking speed was obtained by dividing the lines (cm) a fly crossed (distance walked, *d*) by time, in sec, it spent in activity (activity period, *a*), during the 90 sec assay period. For some flies, both "*d*" and "*a*" were found to be zero. Such individuals were excluded while calculating population mean of *s*. Finally, the horizontal assay was used to extract four locomotor parameters, namely, activity period, distance walked and walking speed. The semi-manual climbing assay described above was validated using DIAS. TD was measured using DIAS, by video recording flies in the treatment vials with media containing 16 mg/ml PTZ. TD is the net path length divided by the total path length. This gives 1.0 for a completely straight path and a smaller value for a meandering path.

### Microarray expression profiling

Total cellular RNA was isolated from fly heads belonging to five biological replicates, four for microarray profiling, and one for real-time PCR. Each replicate represented four independent sets of control and treated flies. Each independent set consisted of eight vials, four each of NF and PTZ, treated in parallel. Similar design was followed for control NF-NF control microarray experiment. Thirty flies were treated in each vial. Heads of 120 flies were pooled for isolating each RNA sample. Flies were frozen in 50 ml falcon tubes in liquid nitrogen. Two cooled sieves were arranged such that the larger sieve (mesh size 850 mm) was placed on top of the smaller one (mesh size 355 mm). Frozen flies were shaken 4–5 times in the falcon and poured onto the top sieve. The flies were brushed gently with a paint brush till all heads were sieved out on the bottom sieve. Bodies that remained on the top sieve were discarded and heads were collected in cryovials and kept frozen at -80°C. Total RNA was isolated from frozen fly heads using TRI REAGENT (Sigma), according to the manufacturer's protocol. RNA was quantified using spectrophotometer. RNA quality was checked by running 1% agarose gel.

Microarray -cDNA Synthesis Kit, -Target Purification Kit, and -RNA Target Synthesis Kit (Roche) were used to generate labeled antisense RNA. Starting with 10 μg of total cellular RNA, Eberwine method (kits from Roche) was used to generate cDNA and thereafter Cy^3 ^and Cy^5 ^(Amersham) labeled antisense RNA. The Cy^3 ^and Cy^5 ^labeled aRNAs (control and treated) were pooled together and precipitated, washed, air-dried, and dissolved in 18 MΩ RNAase free water (Sigma). The recovery of labeled aRNAs was checked using spectrophotometer and agarose gel electrophoresis. A total of 16 microarrays (12Kv1, CDMC) were hybridized, four each for NF-NF control (0 hr); NF-PTZ, 12 hrs; NF-PTZ, 2^nd ^day; NF-PTZ, 7^th ^day. Out of four, two slides were dye-swaps. Hybridization solution was prepared by mixing hybridization buffer (DIG Easy Hyb, Roche), 10 mg/ml salmon testis DNA (0.05 mg/ml final concentration, Sigma) and 10 mg/ml yeast tRNA (0.05 mg/ml final concentration, Sigma) and added to the labeled product. This mixture was denatured at 65°C and applied onto cDNA microarray slides. The slides were covered by lowering down a 24 × 60 mm coverslip (ESCO, Portsmouth, USA). Hybridization was allowed to take place in hybridization chamber (Corning) at 37°C for 16 hrs. After hybridization, coverslips were removed by submerging the slides in a solution containing 1× SSC and 0.1% SDS at 50°C. Slides were washed (three times for 15 minutes each) in a coplin jar at 50°C with occasional swirling and then transferred to 1× SSC and washed with gentle swirling at room temperature (twice for 15 minutes each). Finally, slides were washed in 0.1× SSC for 15 minutes and then liquid was quickly removed from the slide surface by spinning at 600 rpm for 5 minutes. Slides were scanned at 10 μm resolution using GenePix 4000A Microarray Scanner (Molecular Devices), and the images preprocessed and quantified using Gene Pix Pro 6.0 (Molecular Devices).

### Statistics

Unless mentioned otherwise, pair-wise Student's *t*-test, two-tailed, heteroscedastic, uncorrected, was performed for behavioral analysis. In microarray gene expression analysis, ratio based data normalization and selection of features were performed using Acuity 4.0 (Molecular Devices). All Spots with raw intensity less then 100 U and less then twice the average background was ignored during normalization. Normalized data was filtered for the selection of features before further analysis. Only those spot were selected which contained only a small percentage (< 3) of saturated pixels, were not flagged bad or found absent (flags > 0), had relatively uniform intensity and uniform background [Rgn R2 (635/532) > 0.6] and were detectable above background (SNR > 3). Analyzable spots in at least three of four biological replicates performed were retrieved for downstream analysis using SAM 3.0 (Excel Add-In) [38], under the conditions of one class response and 100 permutations. Wherever available, non-CG number genes were converted to CG numbers mainly using BDGP . Gene IDs were converted to FBgn numbers using GeneMerge , before GOTool Box [39] was used to retrieve overrepresented biological processes in up- or down- regulated genes, under the settings, ontology, biological process; mode, all terms; reference, genome; evidence, all-all evidence; species, *D. melanogaster*; GO-stats; statistic test, hypergeometric . DAVID [40] was used for pathway enrichment analysis . *P *values were obtained with or without correction for multiple hypotheses testing, as indicated in the results section. Hypergeometric distribution probabilities for genes and GO processes were calculated assuming population sizes of 10000, approximately the number of unique genes in the arrays, and 4041, a previously estimated number for *Drosophila *[41], respectively.

## Results and Discussion

### Chronic PTZ locomotor model and its validation by AEDs

**Figure 1:**
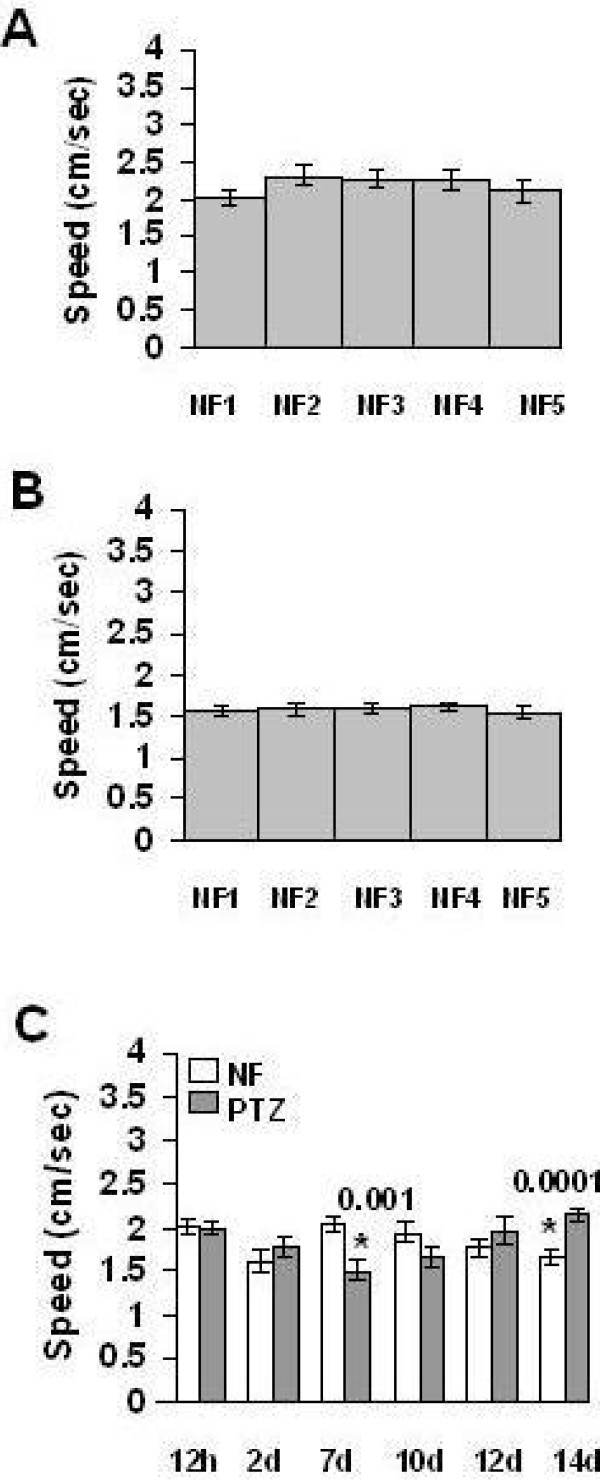
**Chronic PTZ and subsequent withdrawal induced climbing abnormalities**. Mean + S. E. (*n *= 24) of climbing speed in flies used for control experiments and for simulating PTZ induced kindling-like plasticity. In control experiments, five batches of flies were treated with NF for either 7 days (**A**) or 14 days (**B**) before measuring climbing speed. In chronic PTZ experiment (**C**), climbing speed was measured on 12 hrs, 2^nd ^day and 7^th ^day of PTZ treatment and then on 3^rd ^day, 5^th ^day, and 7^th ^day after PTZ withdrawal. For control climbing assay to examine consistency among NF flies housed in different vials (**A **and **B**), one-way ANOVA as well as pair-wise Student's *t*-test, two-tailed, heteroscedastic, uncorrected, was performed. After finding no significant variation by either method, Student's *t*-test was performed as above for chronic PTZ experiment (**C**). * indicates significant difference between control (NF) and PTZ treatment. *p *values (pair-wise Student's *t*-test, two-tailed) are provided over asterisks. For details, see text.

We first explored, through empirical testing, if PTZ could produce proconvulsant-like effect in *Drosophila *adults. Though convulsions were not observed, visual examination indicated that flies treated with 16 mg/ml of PTZ for 12 hrs exhibit hyperkinetic behavior. Software-based analysis of video recordings showed a decreased 'total directionality' in PTZ treated flies compared to control, i.e., flies treated with normal food (NF). Mean + S.E of 'total directionality' was found to be 0.39 + 0.07 (*n *= 11) and 0.19 + 0.05 (*n *= 11) for flies fed with NF and with food containing PTZ, respectively. A significant (*p *= 0.04) difference in 'total directionality' demonstrated that 16 mg/ml PTZ causes flies to take a more circuitous path. As 16 mg/ml was the LC_50 _dose (lethality data not presented), we used half of this dose, i.e., 8 mg/ml, as a sub-hyperkinetic dose for further developing and evaluating a chronic model. Though seizure-like behavior was never observed in flies treated with 8 mg/ml in a chronic manner, we empirically determined a regime in which seven days of chronic PTZ treatment and seven following days of PTZ discontinuation respectively caused a decrease and an increase in startle-induced climbing speed in flies (Figure 1; **for DIAS validation of climbing assay**, see additional file 1). Examining the effect of PTZ on various horizontal locomotor activities and non-locomotor parameters, namely, courtship duration, fertility, and body weight showed that chronic treatment induced decrease and subsequent withdrawal induced increase in climbing speed are specific (**for detailed results**, see additional file 2).

**Figure 2:**
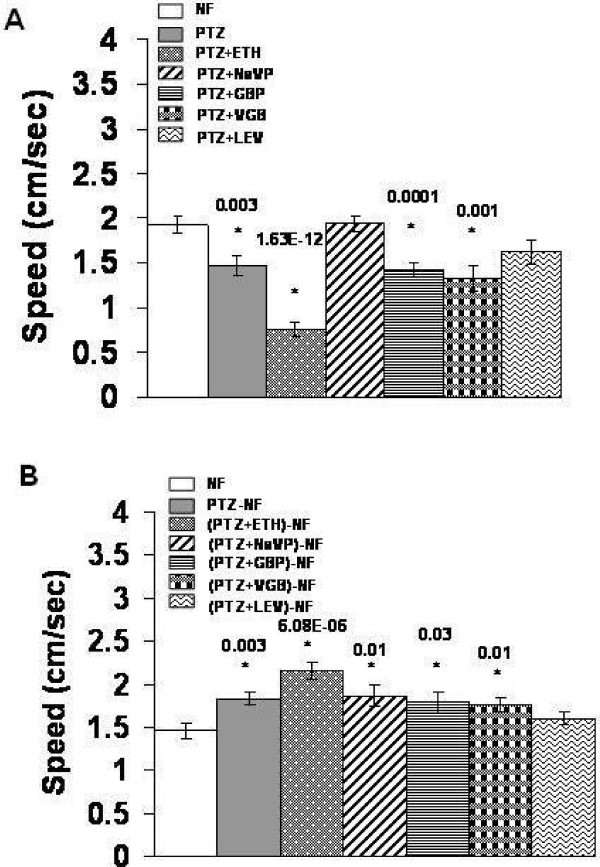
**Effect of AEDs on chronic PTZ and subsequent withdrawal induced climbing speed alterations**. Mean + S. E. (*n *= 24) of climbing speed in flies after (**A**) seven days of combination treatment (**B**) seven days of withdrawal. The difference between PTZ and PTZ+NaVP in **A **and between PTZ and PTZ+LEV in **B **is significant. The difference between PTZ and PTZ+LEV, and between PTZ+NaVP and PTZ+LEV is insignificant in **A**. Notably, comparison of PTZ+LEV in **A **with pooled NF samples in Figure 1A and 1C (7d) showed significant (*p *= 0.0005) difference in climbing speed.

**Figure 3:**
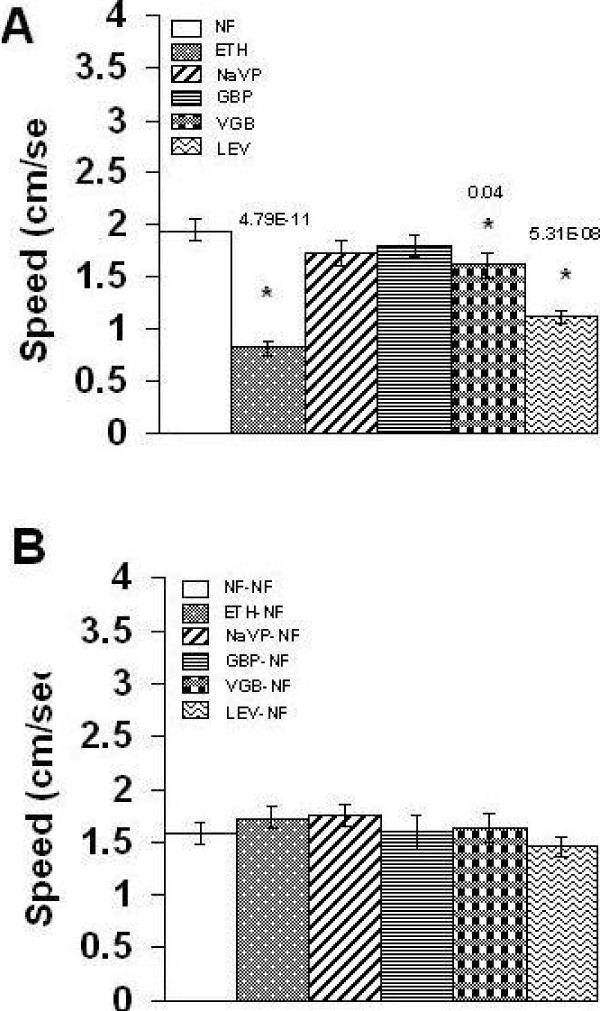
**Effect of chronic AEDs and their subsequent withdrawal on climbing speed**. Mean + S. E. (*n *= 24) of climbing speed in flies after (**A**) seven days of treatment and (**B**) seven days of subsequent withdrawal. For details, see text.

We next examined if AEDs are effective in ameliorating climbing alterations induced by chronic PTZ and withdrawal from chronic PTZ. Concomitant treatment with PTZ and ETH, GBP, VGB, NaVP or LEV for seven days showed the last two AEDs as effective in alleviating PTZ induced climbing speed deficit in flies on 7^th ^day (Figure 2A). NaVP and LEV were thus found to be effective in the fly model. To examine long term effectiveness of AEDs, we concomitantly treated flies with PTZ and an AED for seven days and measured climbing speed seven days after withdrawal of the two drugs. Only LEV, not other AEDs, was found to ameliorate climbing speed increase caused by withdrawal from chronic PTZ (Figure 2B). LEV thus exerted a long term effect. To examine the possibility that the above validation results might have been confounded by AEDs own locomotor effects, we treated flies with AEDs for seven days and measured climbing speed at the end of this chronic treatment as well as seven days after AEDs were withdrawn. ETH, VGB and LEV, not NaVP and GBP, were found to decrease climbing speed at the end of chronic treatment (Figure 3A). Independent speed lowering effect of NaVP and LEV (Figure 3A) could not be considered to confound the observation that these AEDs ameliorate decrease in climbing speed caused by PTZ (Figure 2A). After withdrawal, none of the AEDs were found to cause climbing abnormality (Figure 3B). Cumulatively, the above results validated the PTZ model as predictive of AEDs.

NaVP and LEV, not ETH, GBP and VGB, were found to be effective in our chronic PTZ model of locomotor plasticity. NaVP and LEV are known to suppress both kindling development as well as kindled seizure in rodent models [42,43]. Clinical trials however suggest that LEV, not NaVP, possesses antiepileptogenic activity [9,44,45]. Overall, the profiles of NaVP and LEV in the fly model were consistent with their therapeutic potential. Ineffectiveness of other AEDs in the fly model however suggested that our model is only partly predictive of therapeutic potential. Clinical relevance of the model is also limited by the fact that the drug doses used in developing the model were selected either empirically or based on toxicity.

### Time series of transcriptomic changes underlying chronic PTZ

**Figure 4:**
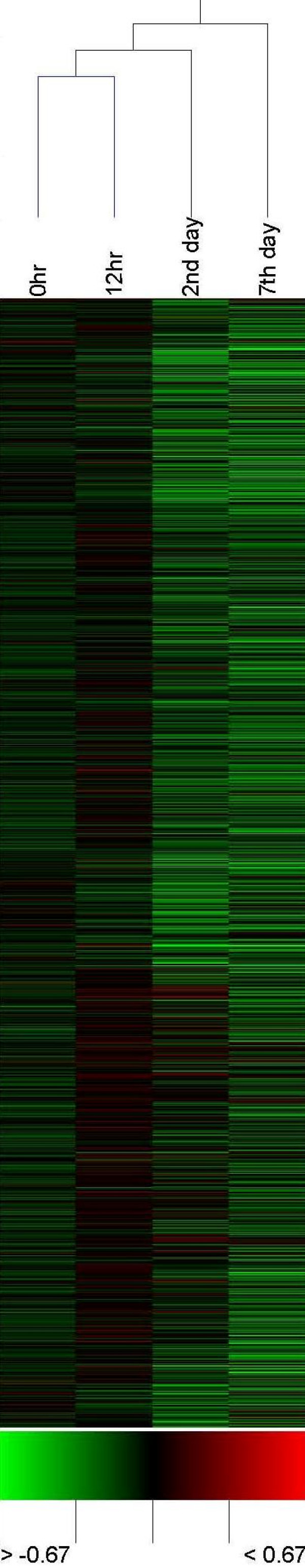
**Time-series of *Drosophila *head microarray gene expression profiles**. Hierarchical clustering of 0 hr, 12 hrs, 2^nd ^day and 7^th ^day expression profiles. City Block similarity metric and average linkage methods were used for clustering of arrays. Each time point represents mean of normalized log_2 _ratio (635/534) of four biological replicates with balanced dye-swaps. The cluster was generated using Acuity 4.0.

We generated expression profiles of fly heads at four time points – 0 hr; 12 hrs of PTZ, the beginning phase; 2^nd ^day of PTZ, the latent phase; and 7^th ^day of PTZ, the behaviorally expressive phase (Figure 4). The full microarray data set has been deposited in the Gene Expression Omnibus  under accession series GSE7037, GSE7120 and GSE7200. Total number of analyzable genes in SAM showed uniformity across time points – 4369, 4637, 5259 and 4297, in that order. At 0 hr, no gene was detected as differentially expressed below 96% FDR. At 12 hrs, 2^nd ^day and 7^th ^day time-points, 23, 2439 and 265 genes were found to be downregulated at 22.76%, 13.74% and 23.03% FDR, in that order (**for gene lists**, see additional file 3). No upregulated gene was detected at above FDRs. Given small number of genes at 12 hrs, we considered these FDRs as the best compromise between uniformity across time points and acceptability in terms of incorporating false positives. We therefore used the above gene lists for all subsequent analysis. Detection of genes which are known to express at a relatively low level in fly brain, *diss *(CG4211), *dnc *(CG32498), *dco *(CG2048), *per *(CG2647), for example [46], as differentially expressed genes in PTZ time series demonstrated that our expression profiling was efficient. Further, a few genes which were used to validate microarray results by real time PCR corroborated the transcriptomic profiles (**for details**, see additional file 4). Notably, significant overlap of genes between adjacent time points [hypergeometric distribution; *p *= 5.3 × 10^-6 ^between 12 hrs and 2^nd ^day, *p *= 1.9 × 10^-22 ^between 2^nd ^and 7^th ^day] demonstrated that our expression profiling was efficient enough to capture the spectrum of transcriptomic alterations underlying chronic PTZ phase.

We first examined if 'seizure' genes already described in *Drosophila *show enrichment in our PTZ induced downregulated genes. Evidence suggests conserved mechanisms between *Drosophila *bang-sensitive mutants and human seizure disorders [20]. Molecular defects in five such mutants involve genes encoding mitochondrial ribosomal protein (CG7925), ADP/ATP translocase (CG16944), the citrate synthase (CG3861), an ethanolamine kinase (CG3525), and an aminopeptidase (CG5518). Prevalence of mitochondrial function disrupting defects in bang-sensitive mutants suggests that impaired energy metabolism may underlie the affected behavior [20]. Importantly, human seizure disorders have been linked to mutations in genes encoding pyruvate carboxylase, and pyruvate dehydrogenase, the two enzymes upstream of citrate synthase, as well as to mutations affecting various steps in the TCA cycle [20]. Our microarrays had all the above bang-sensitive genes spotted except CG7925. Notably, all four spotted seizure genes were downregulated by PTZ. This overlap was statistically significant (hypergeometric distribution, *p *= 0.004).

We next examined enrichment of biological processes in PTZ time series genes. After Bonferroni correction for multiple statistical testing, no GO biological process was observed as overrepresented at 12 hrs. Several processes were however found to be enriched in 2^nd ^and 7^th ^day gene sets. Consistent with unusually large number of genes affected in the latent phase, the 2^nd ^day genes overrepresented a diversity of processes including neuron morphogenesis during differentiation, synaptic transmission, regulation of neurotransmitter levels, neurogenesis, axonogenesis, protein modification, regulation of transcription, axon guidance, actin filament organization etc. (Table 1). In contrast, the behaviorally expressive phase, i.e., 7^th ^day, overrepresented a smaller number of GO processes which included phototransduction, glutamate metabolism, cell communication etc (Table 2). Since entire fly heads including eyes were used for expression profiling, enrichment of phototransduction related processes most likely reflected PTZ's effect on visual perception. In brief, chronic PTZ was found to be associated with downregulation of a large number of genes which were enriched in a diversity of biological processes.

**Table 1:** Overrepresented GO biological processes in genes downregulated on 2^nd ^day of PTZ treatment

**GO_ID**	**TERM**	**P_VALUE**
GO:0000902	cell morphogenesis	1.09E-16
GO:0030154	cell differentiation	1.41E-11
GO:0051179	localization	3.35E-11
GO:0007154	cell communication	7.58E-09
GO:0019226	transmission of nerve impulse	9.01E-09
GO:0006810	transport	1.15E-08
GO:0007267	cell-cell signaling	1.18E-08
GO:0007268	synaptic transmission	1.74E-08
GO:0007399	nervous system development	2.30E-07
GO:0019219	regulation of nucleobase, nucleoside, nucleotide and nucleic acid metabolic process	2.55E-06
GO:0001505	regulation of neurotransmitter levels	3.93E-06
GO:0022008	neurogenesis	5.55E-06
GO:0048666	neuron development	1.70E-05
GO:0030030	cell projection organization and biogenesis	2.57E-05
GO:0048699	generation of neurons	5.83E-05
GO:0031175	neurite development	6.86E-05
GO:0030182	neuron differentiation	0.0001055
GO:0007269	neurotransmitter secretion	0.0003908
GO:0007010	cytoskeleton organization and biogenesis	0.0006173
GO:0016070	RNA metabolic process	0.0006893
GO:0008104	protein localization	0.0008227
GO:0007626	locomotory behavior	0.0009849
GO:0007409	axonogenesis	0.0011489
GO:0006464	protein modification process	0.0027655
GO:0007155	cell adhesion	0.0030866
GO:0045449	regulation of transcription	0.0043652
GO:0043687	post-translational protein modification	0.005309
GO:0048489	synaptic vesicle transport	0.0067834
GO:0016477	cell migration	0.0075502
GO:0006928	cell motility	0.0102359
GO:0007411	axon guidance	0.0141982
GO:0007517	muscle development	0.0142275
GO:0046530	photoreceptor cell differentiation	0.0197214
GO:0001754	eye photoreceptor cell differentiation	0.0297998
GO:0048512	circadian behavior	0.0376882
GO:0008361	regulation of cell size	0.0383864

Some non-specific processes are not shown. For details, see text. *p *values are after Bonferroni correction.

**Table 2:** Enriched GO biological processes in genes downregulated on 7^th ^day of PTZ treatment

**GO_ID**	**TERM**	**P_VALUE**
GO:0019752	carboxylic acid metabolic process	0.0004902
GO:0007602	phototransduction	0.0023181
GO:0009064	glutamine family amino acid metabolic process	0.0034414
GO:0006536	glutamate metabolic process	0.0052215
GO:0007154	cell communication	0.0083634
GO:0006538	glutamate catabolic process	0.0117205

Some non-specific processes are not shown. For details, see text. *p *values are after Bonferroni correction.

**Figure 5:**
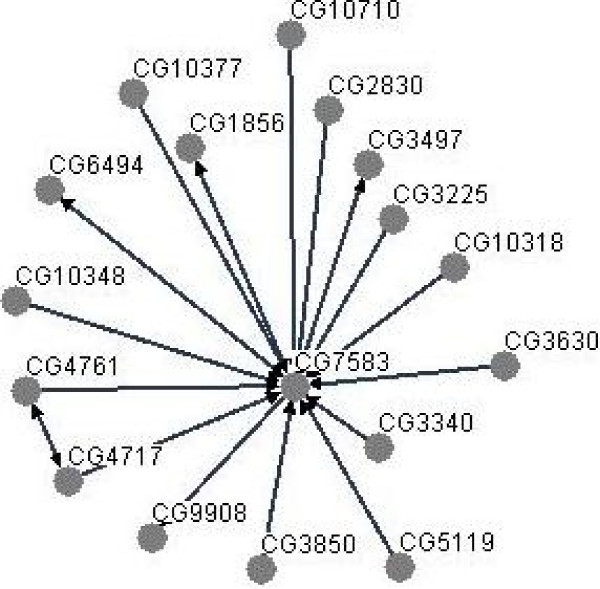
**Overinteraction of CG7583 (CtBP)**. Interacting partners of CG7583 in the network of self-interacting PTZ regulated genes are shown. For details, see text.

For providing direction to the transcriptomic changes, we first overlaid all 2574 genes present in 12 hrs, 2^nd ^day and 7^th ^day sets onto protein interactome map of *D*. *melanogaster *(interaction database BioGRID v 2.0,  visualization software platform Osprey v. 1.2.0, ) which consisted of 7358 genes (vertices) and 24984 connections (edges) [47]. Out of 2574 PTZ regulated genes, 1633 were present in the fly interactome. We retrieved PTZ gene specific subinteractome that consisted of 1633 vertices and 1485 edges. In this subinteractome map, 632 genes were found to be loner, i.e., they did not show any within group interaction. We hereafter focused on within group interaction network which was comprised of 1001 genes and 1485 interactions (**for interacting genes**, see additional file 5). We next examined which of the 12 hrs gene(s) shows statistically significant overinteraction in the network of 1001 vertices and 1485 edges. Among 13 beginning phase, i.e., 12 hrs, genes present in the network, only the C-terminal Binding Protein (CtBP, CG7583) was found to overinteract (hypergeometric distribution after Bonferroni correction, *p *= 0.004; Table 3; Figure 5). CG7583 and its direct interacting partners, a network comprising of 17 genes, showed overrepresentation of various GO processes which were mainly related to transcriptional regulation and cell fate determination (Table 4). Transcriptional alteration was thus identified as the most proximal effect of PTZ. To complete adding direction, we retrieved enriched processes in genes which showed interaction with interacting partners of CG7583 in the self-interacting network of our PTZ genes, and so on, till completion; of 1001 genes, 898 were consumed by this CG7583 centered network. Enrichment of cell morphogenesis and protein import into nucleus; neuron differentiation, neurogenesis, axonogenesis, axon guidance, small GTPase mediated signal transduction, actin filament organization, cell growth, transmembrane receptor protein tyrosine kinase signaling pathway, eye photoreceptor cell differentiation, regulation of cell shape and regulation of cell size; phospholipase C activation and positive regulation of hydrolase activity was observed, in that order (**for complete lists of enriched GO processes**, see additional file 6). Broadly, process enrichment analysis guided by protein interactome suggested that chronic PTZ sequentially downregulates transcription, neurogenesis/axonogenesis/axon guidance and phospholipase C activation. This was consistent with original time series analysis presented above (Tables 1 and 2), except that glutamate metabolism related processes enriched on 7^th ^day (Table 2) were not recovered by interactome guided analysis. Seventh day genes under these categories – CG7145, CG5320 (*Gdh*), CG14994 (*Gad1*), CG1743 (*Gs2*) and CG8430 (*Got1*) – are poorly connected in the entire fly interactome (range, 0–3 interactions). It is likely that non-recovery of glutamate metabolism related processes reflect poor interaction of the genes involved. Cumulatively, the above results suggested that sequential downregulation of gene expression, neurogenesis/axonogenesis/axon guidance, phospholipase C activation and glutamate metabolism characterize our fly behavioral model.

**Table 3:** Interacting partners of 12 hrs genes

**GENES**	**SUBNETWORK INTERACTION**	**NETWORK INTERACTION**	**P_VALUE**
CG11958	1	1	1.768551
CG4799	9	32	0.195
CG8428	1	4	4.55
CG3241	1	3	3.9
CG9238	7	22	2.08
CG11064	1	4	4.55
CG11325	1	2	2.99
CG11963	2	11	3.51
CG9448	1	5	4.94
CG11094	8	46	1.56
CG7583	16	49	0.00455
CG1600	3	4	0.104
CG10078	2	6	1.95

Numbers of partners in the subnetwork of PTZ regulated genes and in the entire interactome network are shown. For details, see text. *p *values are after Bonferroni correction. Note overinteraction of CG7583 (CtBP) in the subnetwork.

**Table 4:** Overrepresented GO biological processes among CG7583 (CtBP) and its direct interacting partners

**GO_ID**	**TERM**	**P_VALUE**
GO:0000122	negative regulation of transcription from RNA polymerase II promoter	8.98E-08
GO:0045934	negative regulation of nucleobase, nucleoside, nucleotide and nucleic acid metabolic process	1.53E-07
GO:0006357	regulation of transcription from RNA polymerase II promoter	1.30E-06
GO:0045892	negative regulation of transcription, DNA-dependent	2.50E-06
GO:0006366	transcription from RNA polymerase II promoter	1.72E-05
GO:0019219	regulation of nucleobase, nucleoside, nucleotide and nucleic acid metabolic process	5.69E-05
GO:0045449	regulation of transcription	0.0003655
GO:0045165	cell fate commitment	0.0004668
GO:0016070	RNA metabolic process	0.0005037
GO:0032774	RNA biosynthetic process	0.0033751
GO:0006139	nucleobase, nucleoside, nucleotide and nucleic acid metabolic process	0.0037604

Partners are limited to the subnetwork of PTZ regulated genes. Some non-specific processes are not shown. For details, see text. *p *values are after Bonferroni correction.

**Figure 6:**
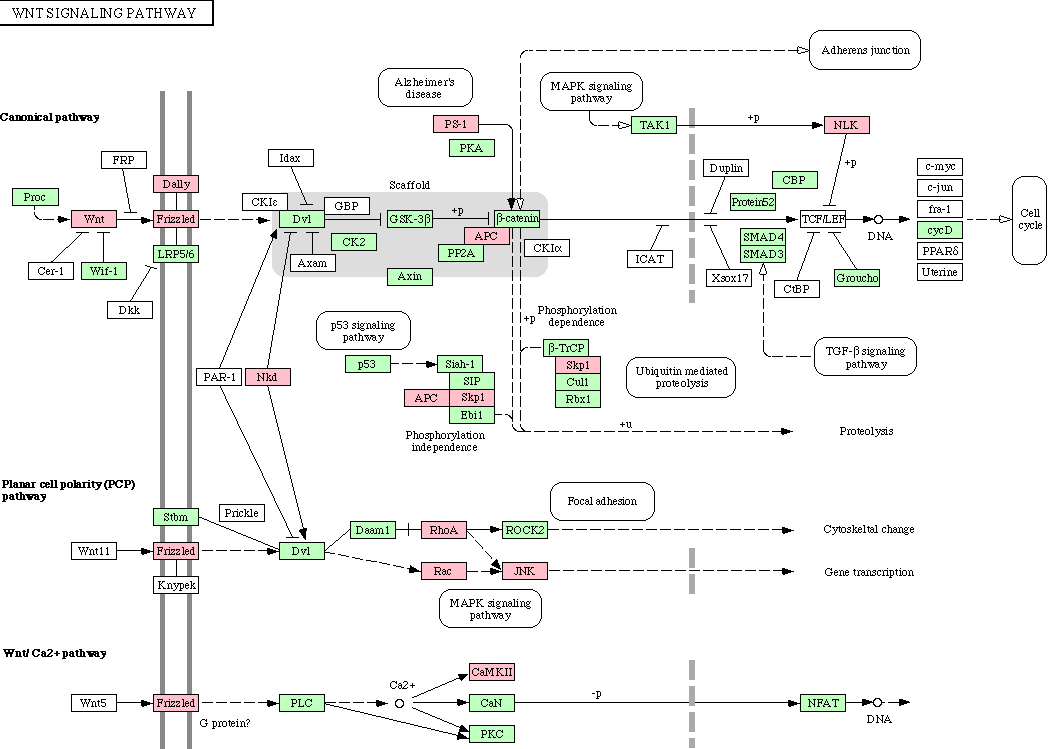
**Enrichment of Wnt signaling pathway**. Pink boxes indicate CG7583 extended network genes that overrepresent one or more GO processes. For details, see text.

**Figure 7:**
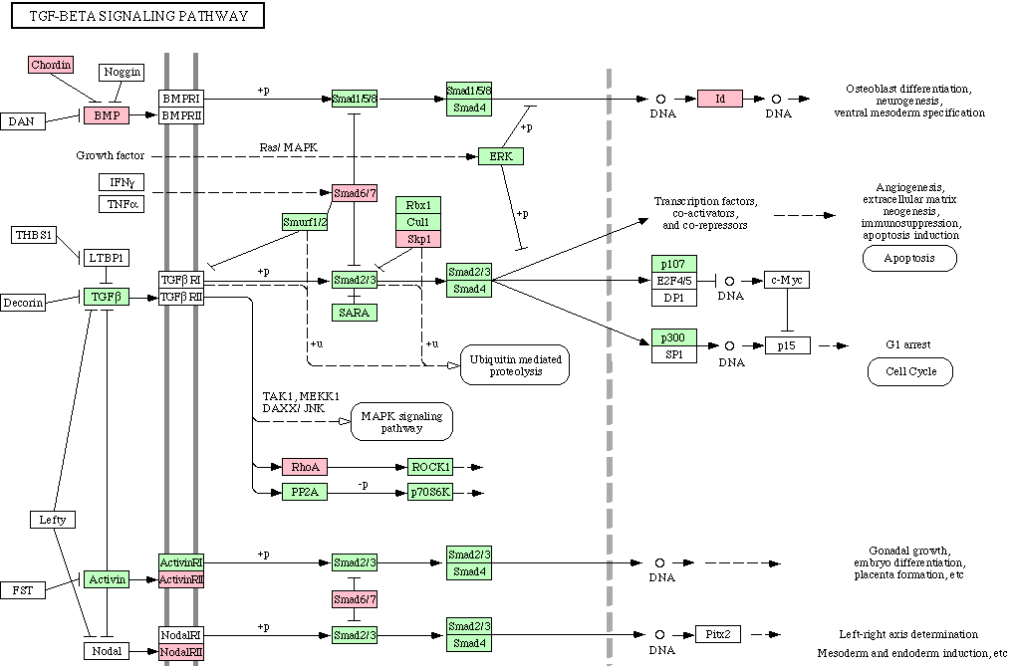
**Enrichment of TGF-beta signaling pathway**. Pink boxes indicate CG7583 extended network genes that overrepresent one or more GO processes. For details, see text.

**Figure 8:**
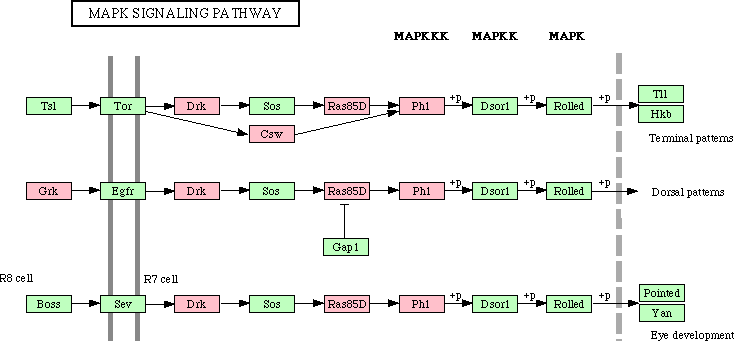
**Enrichment of MAPK signaling pathway**. Pink boxes indicate CG7583 extended network genes that overrepresent one or more GO processes. For details, see text.

**Figure 9:**
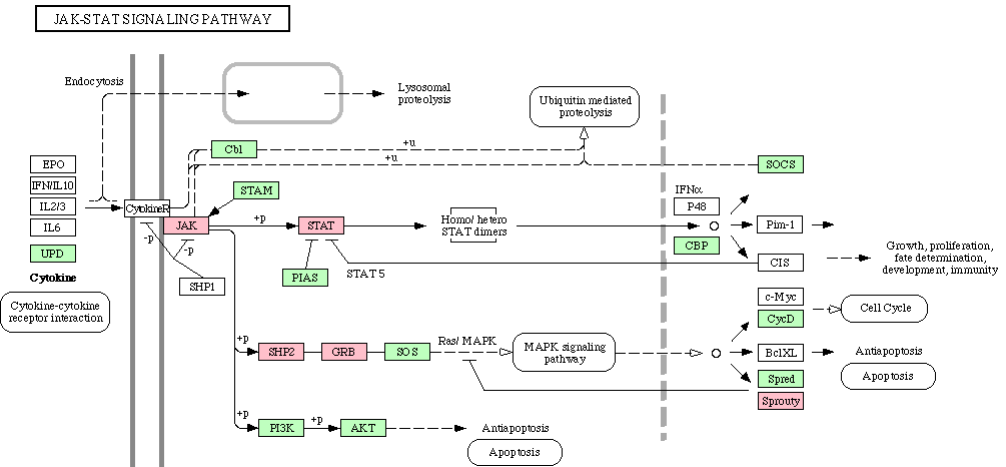
**Enrichment of Jak-STAT signaling pathway**. Pink boxes indicate CG7583 extended network genes that overrepresent one or more GO processes. For details, see text.

**Figure 10:**
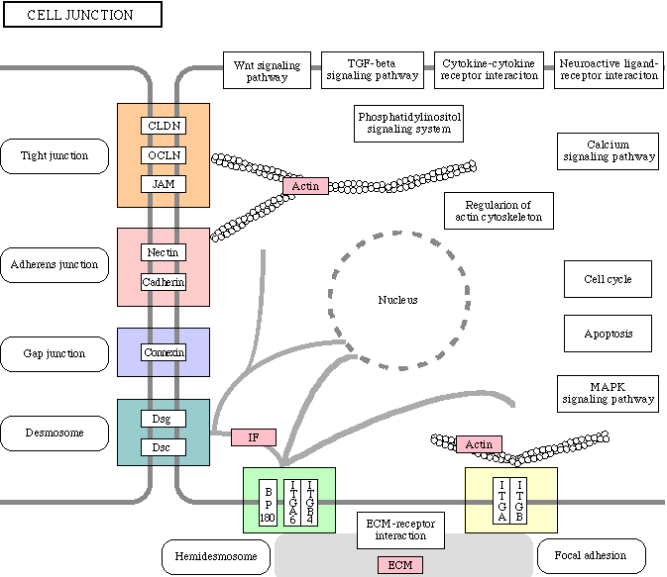
**Enrichment of Cell communication pathway**. Pink boxes indicate CG7583 extended network genes that overrepresent one or more GO processes. For details, see text.

**Figure 11:**
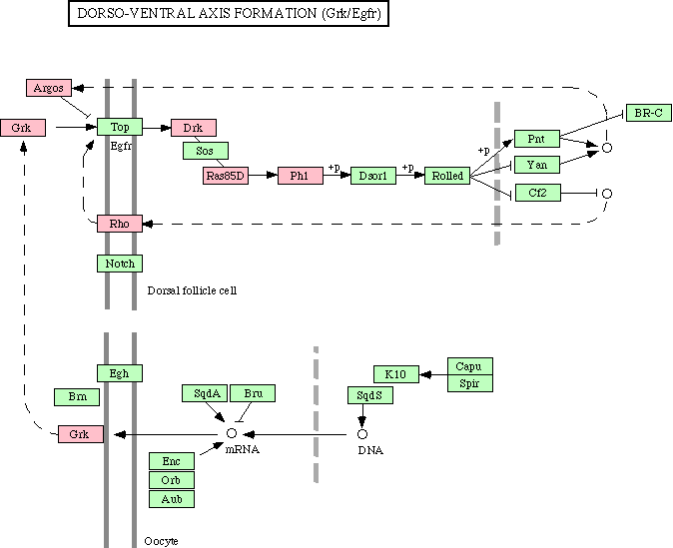
**Enrichment of Dorso-ventral axis formation pathway**. Pink boxes indicate CG7583 extended network genes that overrepresent one or more GO processes. For details, see text.

We next examined enrichment of biochemical and signaling pathways in all 338 genes which were part of enriched biological processes in CG7583 centered network, listed in additional file 6. Although no pathway was found to be enriched after Bonferroni correction for multiple hypotheses testing, our gene set overrepresented various pathways based on unadjusted *p *values (Table 5). Since enrichment of these pathways is consistent with process enrichment analysis presented above – TGF-beta signaling pathway and Wnt signaling pathway are consistent with neurogenesis/axonogenesis/axon guidance, for example – we considered the enriched pathways as significant. Genes showing downregulation in PTZ fly model are indicated in the enriched pathways shown in Figures 6, 7, 8, 9, 10, 11. Remarkably, Wnt signaling which is the most significant pathway in our analysis has previously been implicated in epilepsy [48]. As depicted in Figures 6, Wnt signaling is directly connected to TGF-beta (Figure 7) and MAPK signaling (Figure 8) pathways. Further, one or more of these pathways are also linked to JAK-STAT (Figure 9) and Cell Communication (Figure 10) pathways. Therefore, except Dorso-Ventral Axis Formation (Figure 11) which does not show any direct or indirect connection with Wnt signaling, all other five pathways enriched in our gene set are marked by biological plausibility in epileptogenesis.

**Table 5:** Enriched pathways in CG7583 (CtBP) direct and indirect interacting partners

**CATEGORY**	**TERM**	**P_VALUE**
KEGG_PATHWAY	dme04310:Wnt signaling pathway	0.004599
KEGG_PATHWAY	dme01430:Cell Communication	0.016629
KEGG_PATHWAY	dme04630:Jak-STAT signaling pathway	0.020485
KEGG_PATHWAY	dme04350:TGF-beta signaling pathway	0.026539
KEGG_PATHWAY	dme04320:Dorso-ventral axis formation	0.026539
KEGG_PATHWAY	dme04010:MAPK signaling pathway	0.030198

Genes belonging to overrepresented GO biological processes were used for pathway enrichment analysis. *p *values are unadjusted. For details, see text.

We further examined the relevance of fly model in epilepsy and related neurological and psychiatric conditions through mining reported transcriptomic and proteomic data related to available animal models and human patients. For this, we retrieved rat, mouse and human homologs (FLIGHT, homologene; ) of above mentioned 1001 genes which were downregulated by PTZ and were part of self-interacting network (**for homolog gene lists**, see additional file 7). The homologs were then examined to find out if they overrepresent genes/proteins reportedly downregulated in transcriptomic and proteomic studies on rodent models or human epilepsy (**for gene/protein lists**, see additional file 8). Comparison with individual studies did not reveal statistically significant overlap. Since overlap was also not observed even among reported studies, we pooled down- and up-regulated genes therein separately and matched with our fly gene list. Notably, downregulated genes in fly showed significant match with reported downregulated (*p *= 0.017) but not upregulated (*p *= 0.082) genes. This direction specific overlap demonstrated that our fly model is relevant in understanding epileptogenesis.

## Conclusion

The ultimate goal of systems biology is to delineate and to comprehend the functioning of complex biological systems in such details that predictive models of human diseases could be developed [28]. However, due to immense complexity of higher organisms, systems biology approaches are currently focused on simpler organisms [29,30]. We have described here a behavioral and functional genomic model of chemoconvulsant-induced brain plasticity in *Drosophila*. Although two among five AEDs tested are effective in ameliorating locomotor alteration induced by convulsant drug, flies in our model do not exhibit seizure-like behavior. However, gene expression alterations induced by the convulsant drug show some similarity with expression alterations reported in established rodent models and epileptic patients. Together, these findings suggest that the brain plasticity involved in the fly model may be potentially relevant in understanding mechanisms underlying epileptogenesis to some extent. Chiefly, our model shows downregulation of gene expression and inhibition of neurogenesis/axonogenesis/axon guidance as system level perturbations underlying epileptogenesis-like plasticity. In terms of biochemical pathways, our analysis supports a role of Wnt signaling and other associated pathways in the pathogenesis. Besides epilepsy, AEDs are widely used in treating various neuropsychiatric disorders. Mechanisms of AEDs' long-term action in these disorders are poorly understood. Overlapping pathophysiological mechanisms may underlie epileptogenesis and other neuropsychiatric conditions. Rodent kindling induced by PTZ is a widely used model of epileptogenesis and AED testing. As our fly PTZ model is validated by AEDs, it most importantly offers a unique opportunity for deciphering the mechanisms of long-term action of AEDs that is relevant in various neuropsychiatric conditions. Due to amenability for systems level modeling, candidate gene silencing, small molecule interventions and dietary modifications, for example, can be readily used in our model for generating and testing hypotheses. In brief, the *Drosophila *systems model is expected to be valuable in identifying disease, drug target, biomarker and pharmacogenomic candidates, and in screening of potential therapeutic agents.

## Abbreviations

AED: antiepileptic drug; PTZ: pentylenetetrazole; ETH: ethosuximide; GBP: gabapentin; VGB: vigabatrin; NaVP: sodium valproate; LEV: levetiracetam; NF: normal food; DIAS: Dynamic Image Analysis System; GO: gene ontology; FDR: false discovery rate; TD: Total directionality.

## Authors' contributions

The research was conceived and planned by A.S. Experiments were performed and the data collected by F.M. and P.S., under the supervision of A.S. All authors analyzed the data. A.S. wrote the manuscript, with assistance from F.M. and P.S. All authors read and approved the final manuscript.

## Supplementary Material

Additional file 1 **Validation of semi-manual method for measuring climbing speed by DIAS.** Methods and results (text and figure) pertaining to DIAS based validation of semi-manual climbing speed measurement. Click here for file

Additional file 2 **Effect of PTZ on horizontal locomotor activities and non-locomotor characteristics.** Figures and their description, showing effect of PTZ on locomotor activities and non-locomotor characteristics. Click here for file

Additional file 3 **Genes downregulated at 12 hrs, 2^nd ^day and 7^th ^day of PTZ treatment.** Table listing genes (CG numbers and gene symbols). Click here for file

Additional file 4 **Real time quantitative PCR validation of microarray.** Methods and results (text and figure) pertaining to validation of microarray results by RT-PCR. Click here for file

Additional file 5 **Self-interacting PTZ regulated genes in protein interactome. **Within group interacting partners of PTZ regulated genes. Click here for file

Additional file 6 **Overrepresented GO processes in sequentially interacting genes of partners of CG7583.** Table listing GO IDs, terms, levels, *p *values and genes. Click here for file

Additional file 7 **Homologs of PTZ regulated genes belonging to CG7583 extended network.** Table listing human, rat and mouse homologs of PTZ regulated genes belonging to CG7583 extended network. Click here for file

Additional file 8 **Overrepresentation of epileptogenesis genes in PTZ regulated gene set.** Table listing down- and up-regulated genes in rodent models of epileptogenesis and human epilepsy, and the matching PTZ regulated genes. Click here for file
