# Supplementary material for: A Drosophila systems model of pentylenetetrazole induced locomotor plasticity responsive to antiepileptic drugs
Source: BMC Syst Biol. 2009 Jan 21;3:11. doi: 10.1186/1752-0509-3-11 (PMC2657775; doi:10.1186/1752-0509-3-11)
Supplement: Additional file 1 — Validation of semi-manual method for measuring climbing speed by DIAS. Methods and results (text and figure) pertaining to DIAS based validation of semi-manual climbing speed measurement. [file 1752-0509-3-11-S1.doc]

***Validation of semi-manual method for measuring climbing speed by DIAS***

Movies of flies inside treatment vials were captured in Sony DCR-VX2100-E and transferred from the camera to the inbuilt frame grabber iMovie in an Apple system (PowerMac G5) and compressed for QuickTime using iMovie settings. Movies were opened in DIAS at 25 fps (frames per second) for analysis. First of all, a scale factor was calculated for the movies using a known length. This scale factor (0.018 cm/pixel) was then applied to all the movies. Tracing method used was ‘autotrace by threshold’. Threshold value entered as 150 to eliminate all background and highlight only the object, i.e. fly. For tracing, those frames were selected in which the object traveled the longest continuous distance, i.e., without any path breakage because of jumps or rest. Appropriate entries for scale factor, frame rate and time unit were made in the ‘Edit path file header’ window and speed/directionality of each object was calculated by using ‘compute parameters’ option in DIAS.

**Figure description.** The alteration in fly speed after seven days of chronic PTZ treatment and seven days after PTZ withdrawal i.e. on 7^th^ day and 14^th^ day, respectively, was observed using DIAS. Flies were introduced one by one in the same glass column used for manual measurements, and the vertical climbing for a total length of 8 cm (due the limitation of recording adjustments, 30cm could not be exactly focused in the camera) was video recorded. Path-tracing and the speed calculation were done as explained above, using the ‘compute parameters’ command and frame-by- frame values were averaged to obtain a single value for each object. The difference in the speeds of the two groups compared was found to be significant (Figure given below; *n* = 16, 7^th^ day, *n* = 9, 14^th^ day; * indicates significant difference between NF and PTZ; *p*-value is provided over asterisk). DIAS analysis thus confirmed the difference observed using the semi-manual method. It is notable here that absolute speed between the two methods may differ because of difference in the assays. Whereas semi-manual method calculates climbing speed based on the time spent in activity (dots), DIAS analysis considered the entire traceable path, including momentary rest or jumps, and the time spent.
