# Supplementary material for: A Drosophila systems model of pentylenetetrazole induced locomotor plasticity responsive to antiepileptic drugs
Source: BMC Syst Biol. 2009 Jan 21;3:11. doi: 10.1186/1752-0509-3-11 (PMC2657775; doi:10.1186/1752-0509-3-11)
Supplement: Additional file 2 — Effect of PTZ on horizontal locomotor activities and non-locomotor characteristics. Figures and their description, showing effect of PTZ on locomotor activities and non-locomotor characteristics. [file 1752-0509-3-11-S2.doc]

**Effect of PTZ on horizontal locomotor activities and non-locomotor characteristics.**

(**A**)

(**B**)

(**C**)

**Figure description**. Effect of PTZ on Horizontal locomotor activities. Mean + S. E. (*n* = 24) of (**A**) activity period, (**B**) distance walked, and (**C**) walking speed.

(**A**)

(**B**)

(**C**)

**Figure description**. Effect of PTZ on Non-locomotor characteristics. **(A)** Courtship behavior. A single randomly selected unmated control or treated male was first shifted to an empty vial. A single virgin female of same age, not exposed to any drug at any time, was then introduced in the vial housing the single male. Time at which the female was introduced (*t_a_*) was noted down. Flies were observed throughout courtship. Time at which the male successfully mounted the female (*t_b_*) was recorded. Courtship duration was obtained as *t_b_* - *t_a_*. No difference in courtship duration was observed. Mean + S. E.; *n* = 24, 7^th^ day; *n* = 16, 14^th^ day i.e., 7 days after PTZ withdrawal. (**B**) Fertility. In a vial containing NF, a single, randomly selected, unmated control (NF) or drug exposed (PTZ) male was housed along with a single virgin female, not exposed to any drug at any time, of same age. The parents were discarded after one day and the total number of F1 flies that emerged in each vial was counted as a measure of fertility of the PTZ exposed male flies. No difference in number of flies emerged was observed. Mean + S. E.; *n* = 24, 7^th^ day; *n* = 12, 14^th^ day, i.e., 7 days after PTZ withdrawal. (**C**) Body weight. Flies were over-anesthetized and weighed individually using an analytical balance with a readability of 0.01 mg (Sartorius, model CP225D). Precaution was taken to ensure uniformity in applying the anesthetic diethyl ether. No difference in body weight was observed on either 7^th^ day of chronic PTZ or 14^th^ day, i.e., 7 days after PTZ withdrawal.
