# Supplementary material for: A Drosophila systems model of pentylenetetrazole induced locomotor plasticity responsive to antiepileptic drugs
Source: BMC Syst Biol. 2009 Jan 21;3:11. doi: 10.1186/1752-0509-3-11 (PMC2657775; doi:10.1186/1752-0509-3-11)
Supplement: Additional file 4 — Real time quantitative PCR validation of microarray. Methods and results (text and figure) pertaining to validation of microarray results by RT-PCR. [file 1752-0509-3-11-S4.doc]

**Real time quantitative PCR validation of microarray**

PCR amplification reactions were carried out in an ABI Prism 7700 sequence detection system (Applied Biosystems). RNA of each sample was reverse transcribed into cDNA using High capacity cDNA Archive kit (Applied Biosystems) following manufacturer’s recommendations. All reactions were performed in duplicates using a total of ~50 ng of total RNA per reaction, using custom based gene expression assays, in a 384 well plate. Each assay consisted of two sequence-specific PCR primers and a TaqMan assay-FAM™ dye-labeled MGB probe. 18S rRNA was used as an endogenous control. Data was generated using software SDS 2.1 and C_T_ values were calculated. All genes were detectable under the detection thresholds (C_T_<36) recommended by Applied Biosystems [Bookout *et al.,* 2003]. To compare 18S rRNA and target gene, relative quantification was performed using comparative C_T_ method. Briefly, this comparative C_T_ method involved averaging duplicate samples of each target and endogenous control in both calibrator (i.e. control) and treatment samples [i.e. ΔC_T_ (absolute C_T_ value – endogenous control C_T_ value) and ΔΔ C_T_ (ΔC_T_ for each gene – ΔC_T_ for a common reference gene)]. The fold change was calculated according to the formula 2^−(∆∆CT)^, where ∆∆*C*T was the difference between ∆C_T_ target and the ∆C_T_ calibrator value. ABI gene expression assay IDs used were as follows: Dm01803245_m1 (*GRHR*, CG11325), Dm01805173_m1 (CG9238), Dm01806642_g1 (CG9619), Dm02148936_m1 (*Men*, CG10120), Dm01825396_m1 (*PhKgamma*, CG1830), Dm01846862_m1 (CG33138), Dm01834182_m1 (CG7766), Dm01842786_m1 (*Pdk*, CG8808) and Dm01804635_g1 (*Cyp28*, CG10833).


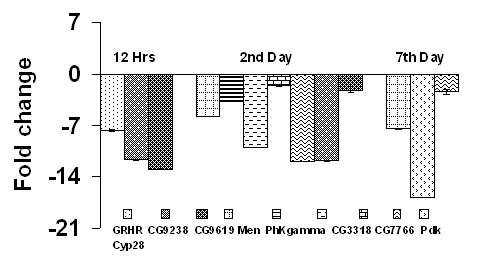


**Figure description**. Validation of microarray gene expression profiling by Real Time PCR. A total of 9 genes were selected for validation of expression profiles representing the three PTZ time-points (12 hrs, 2^nd^ day and 7^th^ day). Downregulation of all 9 genes in microarray was confirmed by RT-PCR.
